# Isolated Inferior Rectus Muscle Entrapment following Endoscopic Sinus Surgery

**DOI:** 10.1155/2018/4620510

**Published:** 2018-07-02

**Authors:** Scott Shapiro, Jamie L. Schaefer, Sumeet Gupta, John Nguyen, Brian Kellermeyer

**Affiliations:** ^1^Department of Otolaryngology, Head and Neck Surgery, West Virginia University, P.O. Box 9200, HSC Room 4520, Morgantown, WV 26506, USA; ^2^Department of Ophthalmology, West Virginia University, P.O. Box 9193, One Medical Center Dr., Morgantown, WV 26506, USA

## Abstract

Orbital complications are known risks of endoscopic sinus surgery (ESS). The lamina papyracea and medial rectus muscle are the most commonly injured structures during ESS. Inferior rectus injury is more rare, with only one reported case of isolated inferior rectus injury in the literature. Guidelines for managing ESS-induced inferior rectus injury do not exist, and delayed intervention and management of adjacent sinuses may affect long-term outcomes such as persistent diplopia and disfigurement. In this report, we present a case of a 67-year-old man with diplopia due to isolated left inferior rectus muscle entrapment and injury from violation of the orbital floor during previous ESS. We postulate that an incomplete maxillary antrostomy contributed to scar band formation and entrapment of the inferior rectus muscle after the orbital floor was violated, and advocate early intervention with a wide, complete maxillary antrostomy if the orbital floor is injured during ESS.

## 1. Introduction

Endoscopic sinus surgery (ESS) is the standard of care for the treatment of medically resistant inflammatory sinus disease, as well as other pathology of, or anatomically related to, the paranasal sinuses. An inherent risk of ESS is injury to the thin bones of the medial orbital wall, and violation of the periorbita can pose significant danger to the extraocular muscle, fat, nerves, and globe. With over 19,000 ESS procedures performed in the US annually, fortunately ophthalmic complications remain rare, occurring in 0.3 to 3% of all procedures [1–4]. Risk factors for ophthalmic injuries include severity of sinonasal disease, surgeon experience, revision surgery, older age patient, and anatomical variations [4, 5]. Most involve injury to the medial rectus muscle via violation of the lamina papyracea, while the orbital floor and adjacent inferior rectus muscle is generally not considered a high-risk area [6]. Although injuries to the inferior rectus muscle have been reported, almost all have been associated with injuries to other orbital structures, usually the medial rectus, with only one reported case in the literature, where injury was isolated to the inferior rectus muscle [7]. There are few guidelines that exist on how to proceed if an orbital floor injury is noticed during ESS, and subsequent management may affect long-term outcomes such as persistent diplopia. In this report of a case, we (1) describe a patient who suffered orbital floor injury and isolated inferior rectus muscle damage from ESS, (2) endoscopically demonstrate the proximity of the orbital floor to the maxillary antrostomy site, and (3) discuss management of the floor and the adjacent sinuses if orbital floor injury is noticed intraoperatively.

## 2. Case Report

**Figure 1:**
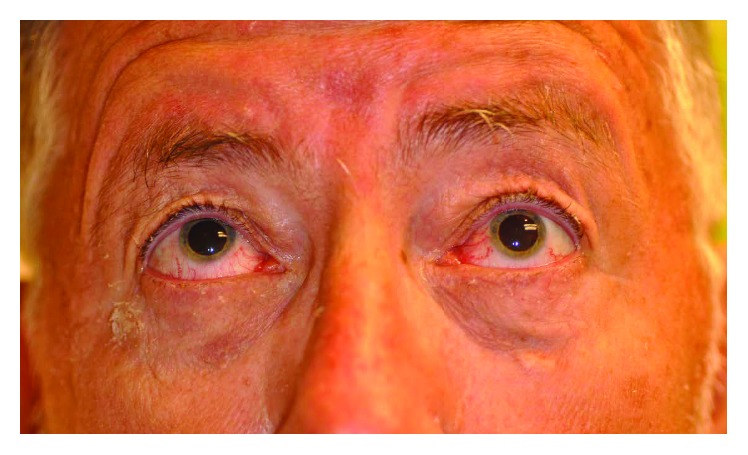
Frontal photograph during superior gaze. Limitation of left superior gaze.

**Figure 2:**
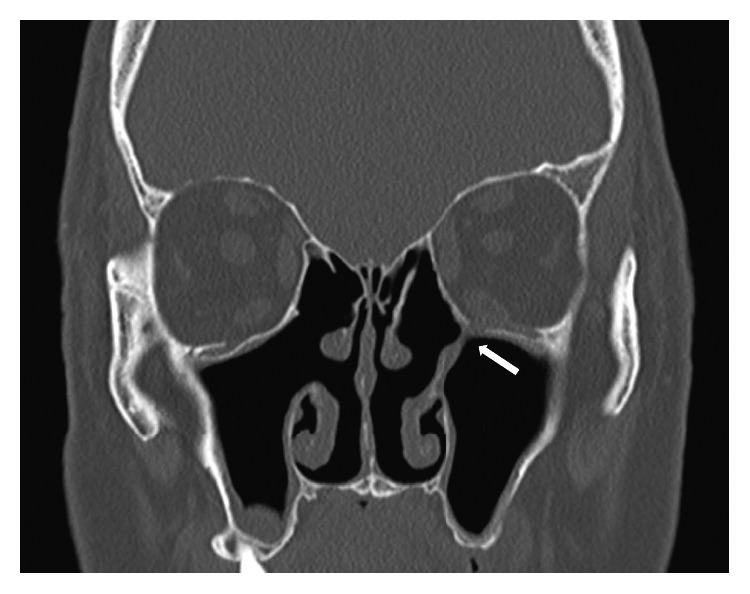
CT scan of the sinuses without contrast, frontal view. 5 mm defect in the posterior medial orbital floor with inferior displacement of the inferior rectus muscle into the defect. Soft tissue band was present from the defect to the remnant of the uncinate process (arrow).

A 67-year-old Caucasian man presented to the ophthalmology clinic with persistent vertical diplopia on left and superior gaze for 8 months. He noticed diplopia upon waking from general anesthesia after an endoscopic sinus surgery which included bilateral ethmoidectomy and medial maxillary antrostomy for chronic sinusitis at an outside facility. Examination revealed restriction of superior gaze of the left eye (Figure 1). A CT scan revealed a 5 mm defect in the posterior medial orbital floor with inferior displacement of the inferior rectus muscle into the defect (Figure 2). A soft tissue band was present from the defect to the remnant of the uncinate process. He was referred to the otolaryngology clinic, and endoscopic examination revealed a small and posterior maxillary antrostomy, with a scar band connecting it to the orbital floor, but no obvious defects in the mucosa or exposed orbital contents.

**Figure 3:**
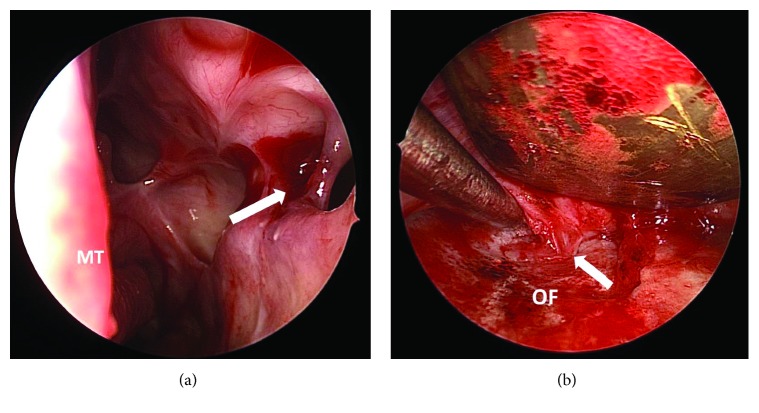
Endoscopic view of left nasal cavity (a) and orbital floor (b). MT, middle turbinate. Arrow denotes scar band tethering the inferior rectus muscle to the sinus mucosa through the defect on the orbital floor (OF).

**Figure 4:**
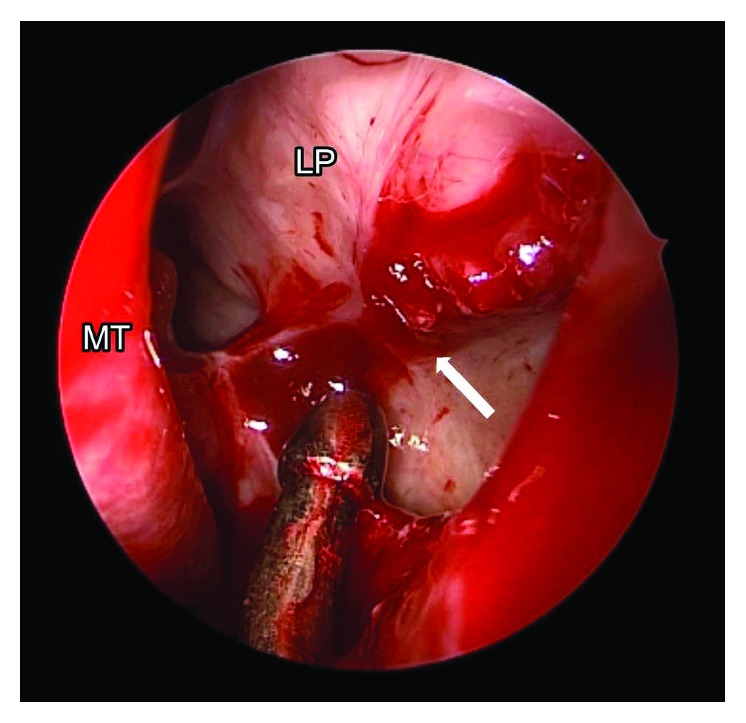
Endoscopic view of the left nasal cavity after revision ESS and uncinectomy. Bulging in nasal mucosa when instrumented through the orbital defect (arrow). LP,  lamina papyracea.

He underwent revision endoscopic sinus surgery to revise the maxillary antrostomy, along with a transconjunctival orbitotomy to release the inferior rectus muscle and repair the orbital floor defect. Intraoperatively, there was a thick scar band tethering the inferior rectus muscle to the sinus mucosa through the defect on the orbital floor (Figure 3). After releasing of the scar band, a round bony defect was observed. From the nasal perspective, there was healthy sinonasal mucosa over the defect, but bulging of that mucosa could be seen when instrumented through the orbit (Figure 4). The forced duction test was free of restriction, and a smooth porous polyethylene implant was used to repair the orbital floor defect. Postoperatively, the left maxillary antrostomy did not develop scarring or restenosis based on surveillance via rigid nasal endoscopy in the office. His diplopia on superior gaze improved but did not resolve entirely 6 months after the revision surgery. He was offered but deferred additional treatment.

## 3. Discussion

Due to its proximity to the surgical field, the lamina papyracea is the most commonly violated boundary of the orbit, and damage to it represents the most common complication of sinus surgery [8–10]. The lamina is at risk during uncinectomy or ethmoidectomy, with the orbital floor at risk during maxillary antrostomy and sinusotomy [11], though the orbital floor is generally not considered a high-risk area during sinus surgery [6]. Although injuries to the inferior rectus muscle have been reported in association with injuries to other orbital structures, most commonly the medial rectus muscle [12], isolated inferior rectus injuries via orbital floor violation are rare with only one reported case in the literature [7]. In their reported case, the orbital defect was significantly larger than in our patient, the patient presented earlier (2 weeks versus our 8 months), and with diplopia on both superior and inferior gaze.

As demonstrated by these two cases, the proximity of the orbital floor to the site of maxillary antrostomy should be a concern during ESS. To prevent this complication, the antrostomy should be made just posterior to the hard lacrimal bone and at the level of the inferior aspect of the ethmoid bulla. Despite the rarity of this complication, it is not difficult to place to an antrostomy slightly posterior and superior which may result in injury to the orbital floor. In our case, it appeared the maxillary antrostomy was halted at this point and not fully opened, thus not connecting with the natural os of the maxillary sinus, as shown in Figure 2.

These two cases highlight some important aspects of management of iatrogenic orbital floor injury during ESS. Guidelines do not exist expressly for this entity, but to some degree, we may rely on guidelines from management of traumatic orbital fractures, which suggest emergent intervention if entrapment is present [13–16]. In ESS-induced injury to the orbital floor, management is complicated by the adjacent sinuses, which may be inflamed due to chronic sinusitis and/or surgery, and may be more likely to develop scarring, which occurred in our patient. We speculate that during the previous surgery, the orbital floor injury was noticed and the surgeon chose to not to complete the antrostomy for fear of causing further injury in this area. It is unknown whether persistent diplopia was the result of direct injury, subsequent scar formation, or both; however, this case does emphasize that if an orbital floor injury is made during antrostomy, and there are no acute complications such as orbital hemorrhage or hematoma, the maxillary antrostomy should be completed to prevent postoperative synechiae, which can contribute to postoperative orbital dysfunction. It may be beneficial to repair the orbital defect at the time of surgery if it is noticed. If it is not noticed intraoperatively, a patient who awakens with diplopia after ESS should elicit a high suspicion for orbital injury, and reexploration or imaging is considered. Delayed care likely contributed to persistent diplopia after surgical repair of the maxillary antrostomy and orbital floor. Perhaps, if our patient more urgently underwent ophthalmologic consultation, with subsequent orbital floor repair and revision sinus surgery, persistent diplopia could have been prevented.

In summary, the orbital floor is in close proximity to surgical field during maxillary antrostomy. Although very rare, orbital floor injury can result from a posteriorly or superiorly placed antrostomy. If orbital floor injury occurs, the floor should be repaired and the antrostomy should be completed to prevent postoperative synechiae which may contribute to postoperative orbital dysfunction.

## Supplementary Materials

Supplementary Materials Supplemental file 1: video version of Figure 4, endoscopic view of left nasal cavity after revision ESS, and uncinectomy. Bulging in nasal mucosa when instrumented through the orbital defect (arrow). LP,  lamina papyracea.
